# Editorial: Pathological livers in the surgery of hepatic resections and liver transplantation

**DOI:** 10.3389/fmed.2022.1072093

**Published:** 2022-11-15

**Authors:** Carmen Peralta, Arani Casillas-Ramirez

**Affiliations:** ^1^Institut d'Investigacions Biomèdiques August Pi i Sunyer (IDIBAPS), Barcelona, Spain; ^2^Hospital Regional De Alta Especialidad de Cd. Victoria, Ciudad Victoria, Mexico; ^3^Facultad de Medicina e Ingeniería en Sistemas Computacionales Matamoros, Universidad Autónoma de Tamaulipas, Ciudad Victoria, Mexico

**Keywords:** liver transplant, ischemia-reperfusion, partial hepatectomy, liver graft, marginal donor

Most organs (80%) submitted to liver transplantation (LT) originate from brain death (BD) donors (1) and 4 to 20% from circulatory death (CD) donors (2). CD and BD cause important hypoperfusion in the mesenteric microcirculation and warm hepatic ischemia, that result in injurious effects on liver grafts used for transplantation (3).

There are two categories of marginal livers: livers with a high risk of impaired function (i.e. elderly or steatotic donors), and grafts entailing the risk of infection or malignancy for the recipient (4). In the coming years, there will be a need to use liver grafts from donors with such pathologies to reduce the waiting-list for transplant (5, 6). It is also foreseeable that the prevalence of metabolic pathologies (that affect negatively the post-operative outcomes) will increase in patients submitted to hepatic resection (7).

Therapeutic strategies are urgently required to reduce the deleterious effects of BD and CD on liver grafts, the poor tolerance of livers with different pathologies to ischemia-reperfusion (I/R) injury, and regenerative failure in resections and LT (8). Despite numerous studies, the impact of each extended criteria donor variable on graft function or recipient survival is still under investigation because of contradictory results (9). Moreover, molecular aspects of the harmful effects of BD and CD are poorly described (2, 10, 11). Discussions on these aspects are the basis to develop targets to improve the post-operative outcomes and to reduce the waiting-list for transplant when a pathological liver undergoing transplantation from deceased donors or resection occurs. As LT is an emergency surgery, advances about quick and non-invasive tools to determine the degree of steatosis and the presence of liver pathologies in the donor, prior to implantation in the recipient, are required.

In addition to being associated with liver resections and LT, I/R injury also occurs in other situations, such as haemorrhagic shock (12). Based on our experience, the signaling mechanisms involved in each clinical situation in which hepatic I/R injury inherently occurs are different, and this fact means that different therapeutic strategies have to be applied depending on each clinical context of I/R (13, 14). So, in clinical hepatic I/R, should the option of personalized medicine be considered? And if this is the case, would it be easy to transfer it to the clinical context?

Before thinking about transferring the results that have been obtained at the experimental level to the clinical field, it is also worth questioning: the numerous signaling pathways reported, do they really mean that in hepatic I/R injury are there multiple mediators and mechanisms involved? Or, are the considerable mechanisms reported the result of the great diversity of experimental models used to study this pathological condition? For instance, to study the underlying pathways involved in warm hepatic I/R, researchers have used a wide variety of factors that influence the post-operative outcome of I/R injury: various ischemia and/or reperfusion times, and the percentage of hepatic ischemia, to name a few. In LT, the same situation also occurs (15).

Another important consideration that must be taken into account is that some studies establish the usefulness of a drug in living donor LT model, and then it is inferred that the same drug can be effective in LT from cadaveric donors. However, the literature presents solid evidence of therapeutic strategies that work in certain transplant conditions but that are useless or even harmful when changing some experimental I/R conditions, or the type of liver graft (16–19). As can be seen, if experimental models that best mimic what happens in clinical practice are not used, preclinical investigations will be of little use, and they will make it difficult to understand hepatic I/R injury.

The articles presented in this issue show advances in the knowledge of new signaling mechanisms involved in hepatic I/R injury that could be useful therapeutic targets to be studied in greater depth and in the medium term, and perhaps applied in the future to improve the clinical liver surgery (Mao et al.). An investigation is included in this issue indicating the advantages of using acellular room temperature machine perfusion to improve the viability of liver grafts recovered from extended criteria donors but the logistical difficulties and costs involved in the use of perfusion machines are known (Abraham et al.). An alternative to reduce waiting lists are therapeutic strategies based on tissue restoration, for which extracellular matrix scaffolds are currently being evaluated and, in this sense, studies are being carried out on the biocompatibility and rejection of synthetic and natural scaffolds as an alternative to LT. In the issue, results are presented demonstrating that the xenoimplant of collagen matrix scaffold is a good niche for hepatocytes, with no rejection, and does not affect liver function tests, thus indicating that this biomaterial could be useful in regenerative medicine for liver diseases (Martinez-Castillo et al.). It is important to continue in the future with studies that determine if this material is equally effective in liver I/R, and in pathological livers (steatosis or diabetes, for example) since regeneration can be affected by this type of pathology (20, 21). The detrimental effect of various conditions on liver viability is also supported by other research included in this issue that describes several factors correlated with the patient and the surgery that directly influence the success of human hepatocyte isolation. For instance, malignant disease, ischemia associated with resections, and male gender among other factors, were associated with lower hepatocyte viability and cell isolation yields (Solanas et al.).

Predicting the clinical post-transplant results is a valuable tool if grafts from extended criteria donors are to be used in the future and satisfactory post-operative outcomes are to be obtained. In this sense, the issue presents an investigation that discusses a predictive model aimed at avoiding donor risk factors in high-MELD score recipients (Yang et al.). Along the same line, a research paper is also presented to explore the relationship between pretransplant intrahepatic proteins such as HO-1, TNF, and the incidence of early allograft dysfunction, thus predicting clinical recipient and graft survival prognosis (Wei et al.). So, does this occur in all types of cadaveric donors? And, what about living donors? The editors believe that further research in this direction may hold great promise for improving the clinical outcomes of LT.

## Author contributions

Both authors listed have made a substantial, direct, and intellectual contribution to the work and approved it for publication.

## Funding

This research has been funded by Spanish “Ministerio de Ciencia” (Grant number: PID2021-123123OB-I00).

## Conflict of interest

The authors declare that the research was conducted in the absence of any commercial or financial relationships that could be construed as a potential conflict of interest.

## Publisher's note

All claims expressed in this article are solely those of the authors and do not necessarily represent those of their affiliated organizations, or those of the publisher, the editors and the reviewers. Any product that may be evaluated in this article, or claim that may be made by its manufacturer, is not guaranteed or endorsed by the publisher.
